# Dynamics of Capillary Lactate Levels in Patients with Out-of-Hospital Cardiac Arrest

**DOI:** 10.3390/medicina59111989

**Published:** 2023-11-11

**Authors:** Vitka Vujanović, Vesna Borovnik Lesjak, Dušan Mekiš, Matej Strnad

**Affiliations:** 1 Center for Emergency Medicine, Prehospital Unit, Community Healthcare Center, Cesta Proletarskih Brigad 21, 2000 Maribor, Slovenia; vesnick@gmail.com (V.B.L.);; 2 Department of Anaesthesiology, Intensive Care and Pain Management, University Clinical Centre Maribor, Ljubljanska ul. 5, 2000 Maribor, Slovenia; dmekis.dm@gmail.com; 3 Faculty of Medicine, University of Maribor, Taborska 8, 2000 Maribor, Slovenia; 4 Emergency Department, University Clinical Centre Maribor, Ljubljanska ul. 5, 2000 Maribor, Slovenia; 5 Department of Emergency Medicine, Faculty of Medicine, University of Maribor, Taborska 8, 2000 Maribor, Slovenia

**Keywords:** cardiopulmonary resuscitation, out-of-hospital cardiac arrest, lactate, return of spontaneous circulation

## Abstract

*Background and Objectives*: An effective strategy for cardiopulmonary resuscitation should be based on tissue perfusion. Our primary aim was to determine the association between capillary lactate values and initial rhythm as well as the probability of the return of spontaneous circulation in out-of-hospital cardiac arrest patients. *Materials and Methods*: This prospective observational cohort study included all patients with non-traumatic out-of-hospital cardiac arrest, older than 18 years, resuscitated by a prehospital emergency medical team between April 2020 and June 2021. Capillary lactate samples were collected at the time of arrival and every 10 min after the first measurement until the time of the return of spontaneous circulation (ROSC) or, if ROSC was not achieved, at the time of declaring death on the scene. *Results*: In total, 83 patients were enrolled in the study. ROSC was achieved in 28 patients (33.7%), 21 were admitted to hospital (26.3%), and 6 (7.23%) of them were discharged from hospital. At discharge, all patients had Cerebral Performance Category Scale 1 or 2. Initial capillary lactate values were significantly higher in patients with a non-shockable rhythm compared to the group with a shockable rhythm (9.19 ± 4.6 versus 6.43 ± 3.81; *p* = 0.037). A significant difference also persisted in a second value taken 10 min after the initial value (10.03 ± 5,19 versus 5.18 ± 3.47; *p* = 0.019). Capillary lactate values were higher in the ROSC group and non-ROSC group at the time of restored circulation (11.10 ± 6.59 and 6.77 ± 4.23, respectively; *p* = 0.047). *Conclusions*: Capillary lactate values are significantly higher in patients with a non-shockable first rhythm in out-of-hospital cardiac arrest (OHCA). There is also a significantly different rise in capillary lactate levels in patients with ROSC.

## 1. Introduction

Despite all of the scientific research and technological advances, sudden out-of-hospital cardiac arrest (OHCA) remains the leading cause of death around the world [1,2,3]. Most of them occur in the home environment [2,3].

OHCA is a sudden malfunction of the cardiovascular system which leads to a sudden decrease in the perfusion of tissues [2]. The causes of cardiac arrest (CA) are primary or secondary [2]. Myocardial ischemia, cardiac channelopathies, myocardiopathies, and other diseases cause primary cardiac arrest, while secondary cardiac arrest is caused by non-cardiac causes like respiratory arrest, trauma, neurological causes, etc. [2,4].

The underlying cause defines the pathophysiological course of cardiac arrest [2,3,4]. In primary cardiac arrest, blood circulation is usually promptly stopped and, in the beginning, presents with an initial shockable rhythm [2,4]. Ventricular tachycardia (VT) can initially produce a perfusing rhythm but when left untreated progresses to pulseless VT or ventricular fibrillation (VF) [2]. Secondary cardiac arrest is characterized by a longer course of time with worsening cardiopulmonary function and the deepening of hypoxia and hypercapnia until the final cardiac arrest [4].

The duration of reduced tissue oxygenation and tissue hypoperfusion defines the difference between primary and secondary cardiac arrest, also on a cellular level [4,5]. Longer-lasting tissue hypoperfusion due to disease causes lactic acidosis [4,6] which leads to compensatory tachypnea and respiratory alkalosis [7,8,9]. If homeostasis is not achieved, the deepening of metabolic acidosis leads to respiratory failure, which results in secondary CA presented as an initial non-shockable rhythm, and higher levels of end-tidal carbon dioxide (EtCO_2_) and lactate are expected [4,8,10,11,12].

Some studies have shown poor neurological outcomes for patients after the return of spontaneous circulation (ROSC) with high levels of lactate at hospital admission [13,14,15]. A clear association between lactate levels and the prognosis of OHCA still remains to be proven [16].

This study aimed to analyze the dynamics of capillary lactate levels between patients with OHCA regarding initial shockable and non-shockable rhythms during advanced cardiopulmonary resuscitation (CPR).

## 2. Materials and Methods

### 2.1. Emergency Medical Service (EMS)

In Maribor, a two-tiered ambulance system, consisting of advanced and basic life support for emergency patient care, is organized. The first team includes two paramedics and an emergency physician and the second includes two paramedics. All of the units are trained in advanced life support. In cases where a severe medical condition or cardiac arrest is suspected, a unit including a physician is dispatched. The EMS protocols are designed according to the Utstein-style reporting for OHCA.

### 2.2. Study Design

This prospective observational cohort study was conducted in Maribor, Slovenia, and adjacent rural areas encompassing a population of about 200,000 inhabitants. The patients enrolled in the present study were all non-traumatic OHCA patients older than 18 years resuscitated by a prehospital emergency medical team. The resuscitation procedures were performed in accordance with the 2015 and updated 2021 European Resuscitation Council guidelines by an emergency medical team.

Patients who were underage, pregnant, or had traumatic CA, in whom CPR was not initiated or blood samples were not taken, were excluded.

After arrival at the scene, EMS advanced CPR was initiated, and an initial capillary lactate sample was obtained as soon as possible (the average time was 5.8 min after the initiation of resuscitation). After that, capillary lactate samples were taken every 10 min during advanced CPR until the time of ROSC or, if ROSC was not achieved, at the time of declaring death on the scene.

All samples were analyzed on the scene with a strip test and a point-of-care BM-Lactate Cobas (Roche Diagnostics GmbH) machine. The measuring range of the blood samples is between 0.8 and 22 mmol/L. Outside this range, the machine defines the measurement as low or high. For the statistical analysis, levels outside the range were not used. For optimal performance, we complet monthly quality control with the use of BM-Control-Lactate strips.

The primary goal of our study was to analyze capillary lactate values during advanced CPR between OHCA patients with initial shockable (VF and VT) and non-shockable rhythms (asystole, pulseless electrical activity (PEA)) and among patients with ROSC and non-ROSC. In addition, the levels of EtCO_2_ between both previously mentioned groups were also compared. The secondary goal of our study was to determine if there is a correlation between average capillary lactate and EtCO_2_ levels.

### 2.3. Statistical Analysis

The statistical analysis was undertaken with IBM Corp. Released 2019. IBM SPSS Statistics for Windows, Version 26.0. Armonk, NY: IBM Corp. Normality was evaluated with the Kolmogorov–Smirnov and the Shapiro–Wilk tests. Two independent groups were compared by independent samples t-test and Leven’s test for equality of variables. Correlation was assessed with Pearson correlation. The value of *p* < 0.05 was considered statistically significant.

The National Medical Ethics Committee of the Republic of Slovenia approved the study and waived the requirement for any informed consent on the 19th of February 2019 (No. 0120-229/2018/12).

The study was registered at Clinical Trials under the number NCT04571281.

## 3. Results

**Figure 1:**
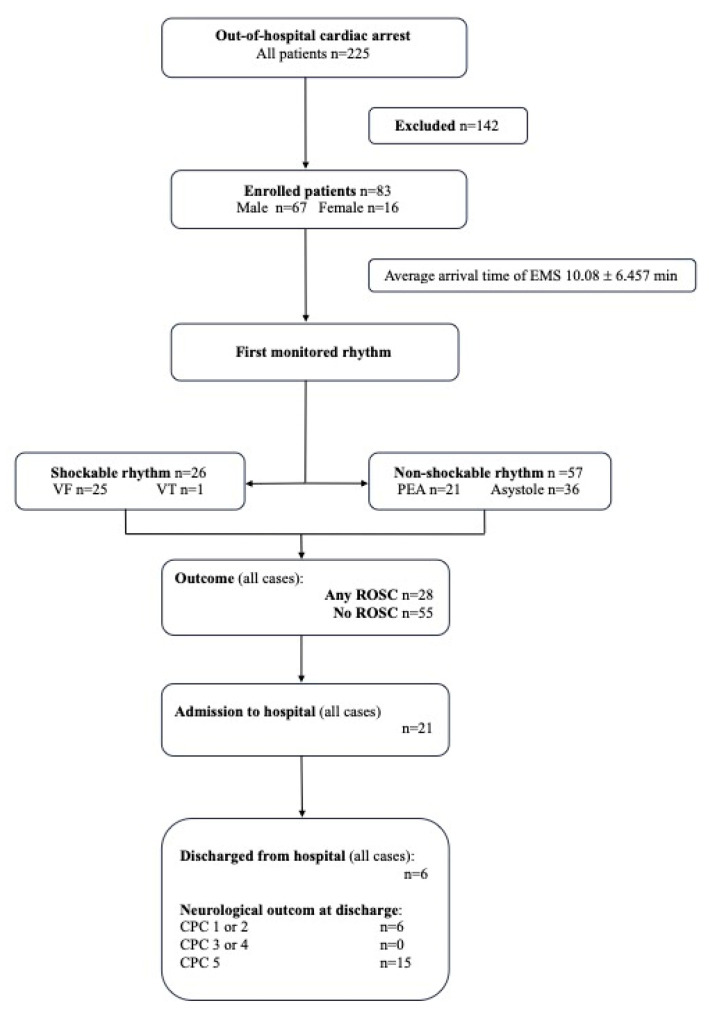
Characteristics of patients with out-of-hospital cardiac arrest (OHCA). Legend: EMS, emergency medical service; PEA, pulseless electrical activity; ROSC, return of spontaneous circulation; VF, ventricular fibrillation; VT, pulseless ventricular tachycardia; CPC, Cerebral Performance Category Scale.

Between April 2020 and June 2021, resuscitation was attempted in 225 patients. In total, 83 patients were enrolled in the study. The remaining 142 patients were excluded from the study if the EMS team achieved ROSC immediately after the first defibrillation, if the EMS team for various reasons failed to perform serial lactate measurements, or if the patient was declared dead on arrival. The median age was 67 years. ROSC was achieved in 28 patients (33.7%), 21 were admitted to hospital (26.3%), and 6 (7.23%) of them were discharged from hospital. Upon discharge, all patients had Cerebral Performance Category Scale (CPC) 1 or 2. Patient characteristics are presented in Figure 1.

Comparison of capillary lactate measurements among the groups based on the first monitored rhythm showed that the initial values were significantly higher in patients with a non-shockable rhythm compared to the group with a shockable rhythm (9.19 ± 4.6 versus 6.43 ± 3.81; *p* = 0.037). A significant difference also persisted in a second value taken 10 min after the initial value (10.03 ± 5.19 versus 5.18 ± 3.47; *p* = 0.019). The values of capillary lactate for both groups are presented in Table 1.

We also compared the values of capillary lactate among the ROSC and non-ROSC groups whereby we found a significantly higher level of the third value of capillary lactate in the ROSC group (11.10 ± 6.59 and 6.77 ± 4.23, respectively; *p* = 0.047). The values of capillary lactate for both groups are presented in Table 2. The average duration of advanced CPR before achieving ROSC was 16.35 ± 9.53 min, while the average duration of advanced CPR before the termination of CPR was 40.13 ± 15.76 min.

**Figure 2:**
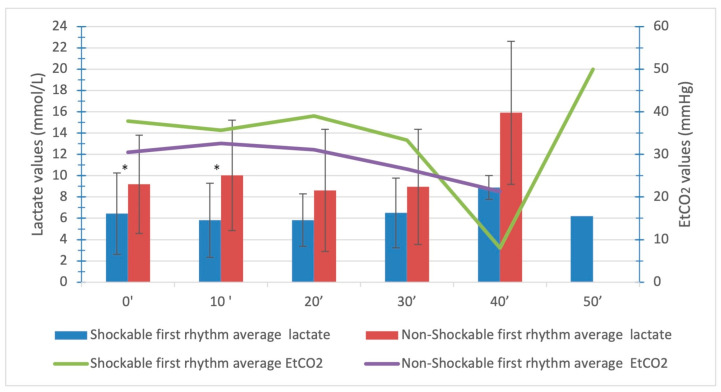
Average capillary lactate and EtCO_2_ levels in a group of patients with OHCA regarding initial rhythm. Legend: *, *p* < 0.05.

Average capillary lactate levels and EtCO_2_ were compared among patients with initial shockable and non-shockable rhythms (Figure 2). In the first ten-minute interval, there was a slight drop in both values in patients with an initial shockable rhythm. The levels of average EtCO_2_ in patients with an initial non-shockable rhythm (asystole, PEA) increased slightly in the first 10 min and progressively decreased until the termination of advanced CPR.

**Figure 3:**
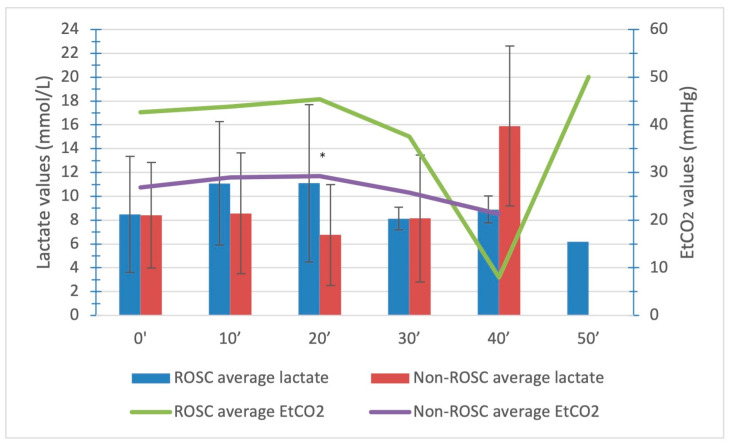
Average capillary lactate and EtCO_2_ levels in the ROSC and non-ROSC groups of patients. Legend: *, *p* < 0.05.

A comparison of capillary lactate levels among the ROSC and non-ROSC groups shows increasing levels at first, with a peak at 20 min in the ROSC group (Figure 3). The non-ROSC group had the highest average level of lactate and the lowest average levels of EtCO_2_ at 40 min after starting advanced CPR. This is also the average point in time when advanced CPR was terminated.

Average capillary lactate levels and EtCO_2_ levels were analyzed and showed no correlation between EtCO_2_ and lactate levels (Table 3).

## 4. Discussion

This study is, to our knowledge, the first to report the dynamics of capillary lactate during advanced CPR in OHCA in the prehospital setting. Comparison of capillary lactate measurements among patients with OHCA showed significantly higher average initial values of capillary lactate in patients with a non-shockable rhythm, which also persisted in a second value taken 10 min after the initial one. When comparing the ROSC and non-ROSC groups, we found that the third average capillary lactate measurement taken 20 min after the initial measurement was significantly higher in patients with ROSC. We found no correlation between EtCO_2_ and the levels of capillary lactate among the same compared groups.

The role of lactate as a biomarker of mortality and predictability of ROSC has been discussed for decades now [13,17,18,19,20]. Stewart et al. published an article emphasizing the importance of correcting acidosis in the management of cardiac arrest [21]. Weil et al. [20] wrote that anaerobic metabolism with lacticaemia provides a quantitative measure for the extent to which the organism is deprived of oxygen and suggested that arterial lactate levels measured during the first 10 min of ongoing CPR and one hour after successful CPR correlate with survival. Their findings on lactate showed that arterial lactate lower than 4.5 mmol/L correlated with patient survival especially if the lactate level was below that level one hour after successful CPR [20]. However, if the initial arterial lactate was above 6.5 mmol/L, only a few patients survived [20]. There were no survivors if lactate exceeded 6.5 mmol/L after one hour [20]. The study by Wang et al. [13], in which serum lactate was measured during the first 10 min of advanced CPR in patients with in-hospital cardiac arrest (IHCA), showed a mean lactate level of 9.6 mmol/L. Their study positively associated shockable rhythm and lactate level <9 mmol/L with survival to hospital discharge [13].

The results from our study also confirm similar observations showing that patients with a shockable first monitored rhythm had lower average initial lactate levels.

Other studies associated post-ROSC mean lactate levels in 24 h [15] or effective lactate clearance with lower mortality [22]. Donnino et al. [22] demonstrated that lactate clearance after ROSC was a better predictor of 24 h and overall, in-hospital survival. A study by Oddo et al. [23] demonstrated that median initial lactate levels are lower in survivors, while a study by Adrie et al. [24] related initial lower lactate levels with good neurological outcomes. The last two studies were carried out in the intensive care unit and reported a initial lactate value cut-off point of 3.1 mmol/L for good neurological outcomes in the study by Adrie [24], which is much lower than the median of 8.1 mmol/L reported by Oddo et al. [23], who described blood lactate at admission as the only other variable associated with survival beside time to ROSC and shockable rhythms.

Most of the studies performed on the dynamics of lactate were carried out in the hospital environment after ROSC was achieved and sustained for different time periods depending on the criteria of the study [21,22,23,24,25,26,27,28]. However, a study by Miomiyama et al. [16] included all OHCA patients admitted to hospital and measured initial lactate and pH upon admission. They reported lower lactate levels in patients with favorable outcomes (82 ± 49 vs. 96 ± 41 mg/dL) but they did not reach statistical significance [16].

In our study, average capillary lactate levels were analyzed mostly because it was easier to carry out repetitive measurements in prehospital settings while performing CPR. The average initial capillary lactate level taken on average 5.8 min after arrival was 6.43 ± 3.81 mmol/L for patients with an initial shockable rhythm and 9.19 ± 4.6 mmol/L for patients with an initial non-shockable rhythm.

Our findings are consistent with the pathophysiological differences between primary and secondary CA [4,5,6,29]. Secondary CA is characterized by progressive and global hypoxia with incomplete ischemia [4]. Reduced whole-body hypoxia and/or severe hypovolemia trigger anaerobic metabolism, causing lactic acidosis and directly reflecting cellular hypoxia [6]. The depletion of cellular energy results in CO_2_ tissue production and the accumulation of CO_2_ in alveoli and initiates biochemical cascades that lead to cell damage and ultimately cell death prior to cardiovascular arrest [4,29]. The correction of hypoxia during CPR and after ROSC requires special attention. EMS teams should aim for normoxemia because hyperoxemia and hypoxemia have been associated with higher mortality and poorer neurological outcomes in OHCA patients [30,31].

Çalbay et al. [32] analyzed initial blood gas samples from OHCA patients at admission to the emergency room (ER) and repeated measurements within 5 min after achieving ROSC and found no correlation between the levels of lactate and ROSC. They reported that PCO_2_ was significant in ROSC estimation and could be used with EtCO_2_ to strengthen the estimation of ROSC [32].

EtCO_2_ is used as a predictor of ROSC and as a measurement to guide our management of cardiac arrest out of hospital [11,33,34,35] because of the known relationship between EtCO_2_ and lactate and its usefulness in predicting the probability of ROSC and mortality [4,7,8,9,11,12,33,34,35]. We analyzed both values and found no correlation between EtCO_2_ and the average levels of capillary lactate among the same groups.

Our study found no significant difference among the ROSC and non-ROSC groups of patients regarding initial capillary lactate level. A study carried out in prehospital settings by Tores et al. [36] presented similar results as they also did not observe a significant statistical relationship between initial lactate level and ROSC or neurological recovery but found an association between base excess and outcome.

We did find a significantly higher third (20 min after the initial capillary lactate level) average capillary lactate value in the ROSC group in comparison to the non-ROSC group (11.10 ± 6.59 versus 6.77 ± 4.23 mmol/L). The difference in average capillary lactate coincides with the average time of restored spontaneous circulation. When we add up the average time for the initial capillary lactate sample (5.8 min after the initiation of resuscitation) and the predetermined time points, we find that, at the time, the significantly higher third average capillary lactate value coincides with ROSC (the average time before achieving ROSC was 16.35 ± 9.53 min) for the ROSC group in comparison with the non-ROSC group. These findings suggest that the highest average level of capillary lactate in the ROSC group (Figure 2) can be associated with restored circulation.

Cardiac arrest represents the most severe shock state [37]. The degree of lactic acidosis correlates with the overall decrease in oxygen delivery, the extent of tissue hypoperfusion, and the severity of the disease process [38]. CRP provides an artificial state of perfusion until physiological circulation is restored [39]. ROSC leads to improvement in tissue perfusion and the clearance of lactate from the tissues to the blood.

It is important to know that post-resuscitation care after OHCA (including normoxemia, temperature control, and even ventilatory settings affecting levels of CO_2_ in the blood) significantly affects the outcomes of OHCA patients [30,31,40,41]. However, this was not part of our study, which only analyzed data up to hospital admission.

In summary, this study suggests that initial capillary lactate levels and capillary lactate levels measured 10 min after CRP could be associated with the type of cardiac arrest. Lactate and EtCO_2_ values in concert with other variables could help clinicians define the cause of the cardiac arrest, the duration of resuscitation procedures, and the timing of the termination of resuscitation. This, however, was not the aim of this study. We emphasize the value of a multimodal approach in prehospital CPR decision-making.

### Limitations

This study has several limitations. The study population is small, and thus, the conclusions need to be tested in larger studies. The study was conducted in a single-center setting because of the different types of EMS organizations among cities in Slovenia and it was observational by design. Another limitation is a lack of all measurements per case during advanced CPR. Thirdly, the interval of 10 min could have been shorter and thereby provided more information about the dynamics of lactate during advanced CPR. The study did not include data on post-resuscitation care, which might have influenced the outcomes.

The COVID-19 pandemic interrupted the conduction of the study.

## 5. Conclusions

Capillary lactate measurements among patients with OHCA showed significantly higher average initial values of capillary lactate in patients with a non-shockable rhythm which also persisted in a second value taken 10 min after the initial one. The findings also suggest that the highest average level of capillary lactate in the ROSC group can be associated with restored circulation.

## Figures and Tables

**Table 1:** Initial and serial values of capillary lactate in patients depending on the first monitored (shockable or non-shockable) rhythm.

Patients		N	Mean	Std. Deviation	*p*
Initial value of lactate	Patients with first monitored shockable rhythm	16	6.43	3.81	0.037
	Patients with first monitored non-shockable rhythm	43	9.19	4.61	
Serial value of lactate after 10 min	Patients with first monitored shockable rhythm	10	5.81	3.47	0.019
	Patients with first monitored non-shockable rhythm	38	10.03	5.19	
Serial value of lactate after 20 min	Patients with first monitored shockable rhythm	9	5.83	2.47	0.075
	Patients with first monitored non-shockable rhythm	21	8.61	5.73	
Serial value of lactate after 30 min	Patients with first monitored shockable rhythm	6	6.52	3.27	0.329
	Patients with first monitored non-shockable rhythm	12	8.95	5.40	
Serial value of lactate after 40 min	Patients with first monitored shockable rhythm	2	8.90	1.13	0.259
	Patients with first monitored non-shockable rhythm	3	15.90	6.71	

Legend: N, number of measurements.

**Table 2:** Initial and serial values of capillary lactate in patients with ROSC or non-ROSC.

	ROSC	N	Mean	Std. Deviation	*p*
Initial value of lactate during CPR	Yes	21	8.48	4.86	0.959
	No	38	8.42	4.43	
Second value of lactate after 10 min	Yes	11	11.09	5.17	0.156
	No	37	8.57	5.06	
Third value of lactate after 20 min	Yes	7	11.10	6.59	0.047
	No	23	6.77	4.23	
Fourth value of lactate after 30 min	Yes	3	8.13	0.95	0.998
	No	15	8.14	5.32	
Fifth value of lactate after 40 min	Yes	2	8.90	1.13	0.259
	No	3	15.90	6.71	
Sixth value of lactate after 50 min	Yes	1	6.20		
	No	0 ^a^			

Legend: CPR, cardiopulmonary resuscitation; ROSC, return of spontaneous circulation; N, number of measurements; ^a^, t cannot be computed because at least one of the groups is empty.

**Table 3:** Correlation between EtCO_2_ and levels of lactate.

		Initial Value of Lactate during CPR	2nd Value of Lactate 10 Minutes after Initial Value	3rd Value of Lactate 20 Minutes after Initial Value	4th Value of Lactate 30 Minutes after Initial Value	5th Value of Lactate 40 Minutes after Initial Value	6th Value of Lactate 50 Minutes after Initial Value
EtCO_2_ (mmHg)–first measurement	Pearson Correlation	0.064	−0.113	0.026	−0.347	−0.822	.^c^
	Sig. (2-tailed)	0.647	0.471	0.903	0.225	0.178	
	N	53	43	25	14	4	1
EtCO_2_ (mmHg)-second measurement	Pearson Correlation	0.174	0.051	0.199	−0.231	−0.968	.^c^
	Sig. (2-tailed)	0.263	0.740	0.319	0.427	0.161	
	N	43	45	27	14	3	0
EtCO_2_ (mmHg)-third measurement	Pearson Correlation	0.135	0.137	0.126	−0.251	−0.349	.^c^
	Sig. (2-tailed)	0.538	0.486	0.530	0.386	0.773	
	N	23	28	27	14	3	0
EtCO_2_ (mmHg)-fourth measurement	Pearson Correlation	−0.084	−0.088	0.264	−0.159	−0.329	.^c^
	Sig. (2-tailed)	0.766	0.737	0.324	0.570	0.671	
	N	15	17	16	15	4	1
EtCO_2_ (mmHg)–fifth measurement	Pearson Correlation	0.157	0.044	−0.805	0.044	−0.056	.^c^
	Sig. (2-tailed)	0.899	0.972	0.404	0.972	0.964	
	N	3	3	3	3	3	0
EtCO_2_ (mmHg)-sixth measurement	Pearson Correlation	.^c^	.^c^	.^c^	.^c^	.^c^	.^c^
	Sig. (2-tailed)						
	N	1	0	0	1	1	1

c. Cannot be computed because at least one of the variables is constant.

## Data Availability

Study data that support the findings of this study are available from the authors upon request.
